# Endometriosis presenting with right side hydroureteronephrosis only: a case report

**DOI:** 10.1186/1752-1947-8-420

**Published:** 2014-12-11

**Authors:** Mert Ali Karadag, Turgut Aydin, Ozge Idem Karadag, Huseyin Aksoy, Aslan Demir, Kursat Cecen, Umit Yener Tekdogan, Urfettin Huseyinoglu, Fatih Altunrende

**Affiliations:** 19 Department of Urology, Kafkas University Faculty of Medicine, Paşaçayırı Kampüsü, 36040 Kars, Turkey; 20 Department of Gynecology and Obstetrics, Acibadem Kayseri Hospital, Seyitgazi Mah, Mustafa Kemal Paşa Blv No:1/1A, 38000 Kayseri, Turkey; 21 Department of Gynecology and Obstetrics, Kayseri Military Hospital, Esenyurt Mah, 38000 Kayseri, Turkey; 22 Department of Anesthesiology and Reanimation, Kafkas University Faculty of Medicine, Paşaçayırı Kampüsü, 36040 Kars, Turkey; 23 Department of Urology, Istanbul Bilim University Faculty of Medicine, Mehmetçik Cad., Cahit Yalçın Sok. No: 1 Mecidiyeköy, 34394 Istanbul, Turkey

**Keywords:** Ureteral endometriosis, Ureteral reimplantation, Hydronephrosis

## Abstract

**Introduction:**

Endometriosis can be defined as the presence of endometrial glandular and stromal tissue outside the uterus. Affected sites of endometriosis can even be the urinary tract. Here, we present the case of a 30-year-old woman with right ureteral endometriosis. This case was important due to the unusual localization and no signs of the disease except for hydroureteronephrosis.

**Case presentation:**

A 30-year-old Caucasian woman with para 2 was admitted to our department for right side flank pain, dysuria and suprapubic pain. She had no complaints of vaginal discharge, bleeding or painful menstruation. Her menstrual cycles were normal and lasting for three to four days. She did not have a history of any surgical interventions. A physical examination revealed a right side costovertebral angle and suprapubic tenderness. Laboratory test results including a complete blood count, serum biochemical analysis, urine analysis and urine culture were normal. Urinary ultrasonography showed right side hydroureteronephrosis with renal cortical thinning. We suspected a right ureteral stone obstructing the ureter and a computed tomography scan was performed. The computed tomography scan revealed similar right side hydroureteronephrosis with obstruction of the ureter. No signs of stone were observed on the scan. Retrograde pyelography and diagnostic ureterorenoscopy were performed and they showed a focal stricture with a length of approximately 3cm at the distal ureteral part and secondary hydroureteronephrosis. Open partial ureterectomy and ureteroneocystostomy with Boari flap were performed. The pathologic specimen of her ureter demonstrated intrinsic endometriosis of the right ureter with endometrial glandular cells and stromal tissue.

**Conclusions:**

Clinicians should suspect ureteral endometriosis in premenopausal women with unilateral or bilateral distal ureteral obstruction of uncertain cause. The main goals of the treatment should be preservation of renal function, relief of obstruction and prevention of recurrence.

## Introduction

Endometriosis can be defined as the presence of endometrial glandular and stromal tissue outside the uterus [1]. It is a chronic estrogen-dependant gynecological disease and affects 6 to 10% of reproductive-age women [2]. Although it is an estrogen-dependant disorder, postmenopausal women can also suffer this pathology [3]. Chronic bleeding, endometrial glands and stroma, and inflammation signs are common histologic features that are observed in specimens [2].

Affected sites of endometriosis can be the peritoneum, ovary, rectovaginal septum, even the urinary tract. Urinary tract involvement is approximately 1% [4]. The ratio of invasion rates for bladder, ureter and kidney are 40:5:1. One ureter, commonly the left side, is mostly affected and can potentially lead to loss of renal function by severe hydroureteronephrosis and urinary tract obstruction. Urinary endometriosis has a broad spectrum of symptoms involving abdominal or flank pain, renal colic, hematuria, cyclical dysuria, urgency, frequency and suprapubic pain.

Right ureteral endometriosis is observed less frequently than the left side. This case was important due to the unusual localization and no signs of the disease except for hydroureteronephrosis. In this report, we present a 30-year-old woman with right ureteral endometriosis.

## Case presentation

A 30-year-old Caucasian woman with para 2 was admitted to our department for right side flank pain, dysuria and suprapubic pain. She had no complaints of vaginal discharge, bleeding or painful menstruation. Her menstrual cycles were normal and lasting for three to four days. She did not have a family history or any surgical interventions. A physical examination revealed a right side costovertebral angle and suprapubic tenderness. Laboratory test results including a complete blood count, serum biochemical analysis, urine analysis and urine culture were normal. Urinary ultrasonography showed right side hydroureteronephrosis with renal cortical thinning. We suspected a right ureteral stone obstructing the ureter and a computed tomography (CT) scan was performed. The CT scan revealed similar right side hydroureteronephrosis with obstruction of the ureter. No signs of stone were observed on the CT scan.

After preclinical and radiologic evaluation of the patient, we decided to perform a right side diagnostic ureterorenoscopy (URS) and retrograde pyelography. Retrograde pyelography and diagnostic URS showed a focal stricture with a length of >3cm at the distal ureteral part and secondary hydroureteronephrosis. The stricture site could not be passed with URS and a double J stent was inserted and the operation was terminated. We planned to perform an excision of the affected ureteral segment and a ureteroneocystostomy with Boari flap six weeks later.

Open partial ureterectomy and ureteroneocystostomy with Boari flap were performed. First, we dissected and excised the affected ureteral segment, which was followed by mobilization of the spatulation of the remaining ureteral parts. Then, the bladder was mobilized anteriorly and a Boari flap was created. The flap was pulled to the spatulated ureter without tension. A new 26cm 4.8F double J stent was inserted and the ureter was anastomosed to the apex of the flap with absorbable 4/0 sutures. Control of the anastomosis was confirmed by filling the bladder with 300ml saline and no leakage was observed. A pelvic drain was placed at the end of the procedure.

**Figure 1:**
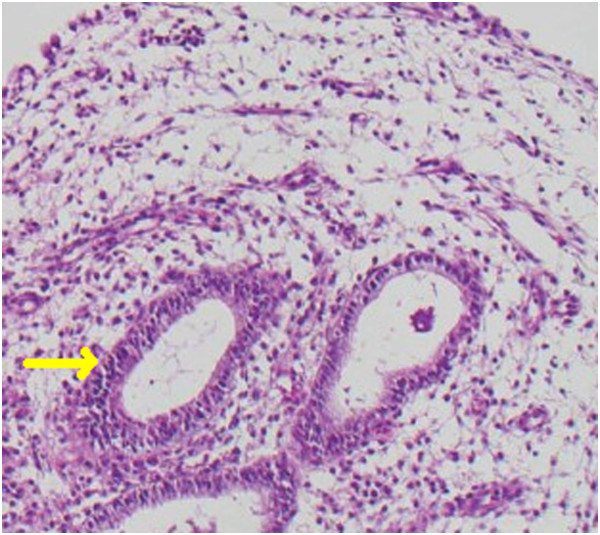
**Arrow showing the endometrial glandular cells and stromal tissue.**

No extravasation was observed from the anastomosis site on the 10th postoperative day and the urethral catheter was removed. We took out the pelvic drain the day after. Our patient was discharged on the 11th postoperative day and the double J stent was removed after six weeks. The pathologic specimen demonstrated intrinsic endometriosis of the right ureter with endometrial glandular cells and stromal tissue (Figure 1). Now, our patient is in the second year of follow-up and has not experienced any signs of recurrence during the last year and control urinary ultrasonographies revealed no evidence of hydronephrosis.

## Discussion

Endometriosis is mostly observed in women between menarche and menopause due to fluctuating levels of estrogen and progesterone, which are required for the stimulation of endometrial tissue [5]. This disease affects approximately 6 to 10% of women of reproductive age [2]. Besides this, this pathology can be observed in the postmenopausal period in an atypical manner that can lead to serious complications [3].

Three types of endometriosis are described according to the morphology and localization: ovarian, superficial peritoneal and deep infiltrative endometriosis (DIE). DIE most commonly invades the rectovaginal space, uterosacral ligaments, bowel or urinary tract [6]. Our case was a DIE because of the right ureteral invasion.

Urinary tract involvement by endometriosis is observed in 1% of women having pelvic endometriosis and the most affected urinary organ is bladder [7]. Ureteral endometriosis (UE) is a rare and usually asymptomatic serious phenomenon that can cause obstructive uropathy unilaterally or bilaterally. This can be observed in minimal or extensive disease: UE is an expression of DIE and is usually related to other endometriosis sites such as rectovaginal space, uterosacral ligaments, bowel tract and parametrium [8]. It is proposed that the distal segments of the ureters, commonly the left side, are more frequently involved due to neighboring the reproductive organs [9]. In our case report, the invaded ureteral segment was also the distal part, but an interesting point was that it was on the right side. The left ureter was normal and no signs of endometriosis were observed. We did not suspect any endometrial tissue during surgery and did not perform any biopsies for the staging of endometriosis.

There are two major pathological types of ureteral endometriosis: intrinsic and extrinsic. Extrinsic type is the most common one and caused by invasion of only the adventitia of the ureter or surrounding connective tissue by endometrial glandular or stromal cells. On the other hand, the intrinsic type of ureteral endometriosis can be defined as involvement of muscularis propria, lamina propria or ureteral lumen with endometrial tissues [10]. Our patient had an intrinsic right ureteral endometriosis, which was confirmed by the pathologic result, with the invasion of the lumen causing focal stricture.

The symptoms of urinary tract endometriosis can be confusing due to its broad spectrum. Albeit only present in 20% of cases, cyclical gross hematuria is pathognomonic for bladder endometriosis [11]. If a woman of reproductive age is having these symptoms with no documented infection, endometriosis should be suspected. Symptoms are especially aggravated during menses, because blood is increased within the invaded organs and this can distend the surrounding tissue or peritoneum [5]. Our case’s symptoms were only right side flank pain, dysuria and suprapubic pain. She had no complaints of dysmenorrhea, dyspareunia or infertility. Also, she did not complain of any cyclical urinary symptoms, suspicious for urinary endometriosis. A vaginal examination should be performed in all patients with suspected endometriosis [12]. She had a normal vaginal examination prior to surgery and in follow-up visits.

Treatment modalities of UE are a dilemma; but the main goals of the therapy should be preservation of renal function, relief of obstruction and prevention of recurrence. Hormonal therapy does not change obstruction secondary to fibrous tissue and adhesions in UE [9]. We did not prefer a hormonal therapy in our patient due to the ureteral obstruction and severe hydroureteronephrosis. If there had been a delay in the treatment of our patient, kidney function might have been lost. Although medical treatment may be effective in some cases of DIE, there is agreement among different experts to remove all visible endometriotic lesions, especially deep endometriotic lesions during surgery [8, 13].

Surgical therapy is mandatory in patients with UE having persistent symptoms and/or hydroureteronephrosis [14]. Laparoscopic approaches like ureterolysis, ureterostomy, distal ureterectomy, ureteral reimplantation or for ureteral strictures secondary to UE can be performed with the advantages of superior exposure, magnified view and easy identification of disease in the peritoneum or pelvis [15].

The surgical modalities of UE may vary according to intrinsic or extrinsic type. Elective ureterolysis by laparoscopic or open approach should be indicated in patients with extrinsic UE, if there is an extrinsic lesion <3cm and/or nonobstructive ureteral involvement [14]. Ureterolysis is contrindicated in patients with intrinsic UE due to high recurrence rate and ureteral stenosis. Treatment of patients having intrinsic UE includes resection of the ureter, if the affected segment is >3cm and below the level of the iliac vessels [9]. We preferred an open partial ureterectomy and ureteroneocystostomy with Boari flap for our case due to intrinsic UE, which involved a >3cm segment in the right distal ureter. Lack of expertise in reconstructive laparoscopic surgery made us prefer the open approach for this patient.

## Conclusions

Clinicians should suspect UE in premenopausal women with unilateral or bilateral distal ureteral obstruction of uncertain cause. The main goals of the treatment should be preservation of renal function, relief of obstruction and prevention of recurrence. Hormonal or surgical therapy choices should depend on the patient’s characteristics and type of UE.

## Consent

Written informed consent was obtained from the patient for publication of this case report and accompanying images. A copy of the written consent is available for review by the Editor-in-Chief of this journal.

## Authors’ information

AK, KC, AD, and UH are assistant professors of urology. TA, OIK, and HA are gynecologists and obstetricians. FA and UYT are associate professors of urology.

## Abbreviations

CT: computed tomography
URS: ureterorenoscopy
DIE: deep infiltrative endometriosis
UE: ureteral endometriosis.
